# Radiomics-based causal machine learning for exploratory treatment-effect estimation of neoadjuvant chemotherapy cycle intensity in osteosarcoma: a proof-of-concept study

**DOI:** 10.1186/s12880-026-02533-7

**Published:** 2026-06-30

**Authors:** Zijie Yuan, Ruoyao Wang, Hao Zhang, Zhixian Zheng, Xiuxiu Zhou, Jingyu Xing, Weihao Zhai, Xinghao Cao, Chenglong Zhao, Taohu Zhou, Li Fan, Cheng Yang

**Affiliations:** 1 Department of Orthopedic Oncology, Changzheng Hospital, Second Military Medical University, Shanghai, 200003 China; 2 Department of Radiology, Changzheng Hospital, Second Military Medical University, Shanghai, 200003 China; 3 Department of Radiology, The 905th Hospital of the Chinese People’s Liberation Army Navy, Shanghai, 200003 China

**Keywords:** Osteosarcoma, Radiomics, Causal machine learning, Individual treatment effect, Neoadjuvant chemotherapy, Treatment-effect estimation, Proof-of-concept study

## Abstract

**Background:**

Radiomics-based modeling has shown promise for characterizing tumor heterogeneity, but its integration with causal machine learning for treatment-effect estimation remains underexplored in osteosarcoma.

**Objective:**

This study aimed to develop a proof-of-concept radiomics-based causal machine learning framework for exploratory estimation of average and individual treatment effects associated with neoadjuvant chemotherapy cycle intensity in osteosarcoma.

**Methods:**

This retrospective single-center study included 34 patients with osteosarcoma who underwent neoadjuvant chemotherapy followed by surgical resection. Radiomic features were extracted from pre-treatment T1-weighted magnetic resonance imaging and combined with baseline clinical variables. Three causal meta-learners—S-Learner, T-Learner, and X-Learner—were implemented to estimate counterfactual survival probabilities under high-cycle and low-cycle neoadjuvant chemotherapy strategies. Average treatment effects and individual treatment effects were derived from the predicted potential outcomes.

**Results:**

The proposed framework enabled estimation of population-level and individualized treatment-effect measures using integrated radiomic and clinical covariates. The estimated average treatment effects differed in magnitude and direction across meta-learners, indicating instability of treatment-effect estimation in this small cohort. Confidence intervals crossed zero for two of the three learners, and model performance metrics were interpreted only as technical indicators of feasibility rather than evidence of generalizable predictive validity.

**Conclusion:**

This study demonstrates the methodological feasibility of combining radiomics with causal machine learning for exploratory treatment-effect estimation in osteosarcoma. Given the limited sample size, retrospective single-center design, treatment-group imbalance, missing outcome information, and uncertainty of causal assumptions, the findings should be regarded as hypothesis-generating rather than clinically actionable. Larger multicenter studies with standardized imaging protocols, adequate event counts, longer follow-up, and prospective validation are required before the translational relevance of radiomics-based causal treatment-effect estimation can be assessed.

**Supplementary Information:**

The online version contains supplementary material available at https://doi.org/10.1186/s12880-026-02533-7.

## Background

Causal inference is fundamental to evidence-based medical research, as it aims to estimate the effects of clinical interventions while accounting for confounding in observational data [1]. Classical causal inference methods, including propensity score–based approaches, instrumental variable analysis, difference-in-differences, and regression discontinuity designs, have been widely applied in medical studies. However, their practical applicability may be constrained by strong methodological assumptions, such as the availability of valid instruments, the need for specific quasi-experimental settings for difference-in-differences and regression discontinuity, and the potential for residual confounding in propensity score–based analyses. These traditional linear regressions or propensity score adjustments typically require a high ratio of events-per-variable (EPV) to ensure robust coefficients, which poses an important limitation when treatment decisions are influenced by complex clinical and imaging-derived factors that are difficult to model using traditional approaches [2–4]. This constraint is particularly critical in small-sample observational settings where a severe deficit in degrees of freedom can cause standard statistical convergence to fail.

In response to these limitations, causal machine learning (CML) has been proposed as an extension of classical causal inference that leverages machine learning to estimate treatment effects from observational data [5]. By modeling potential outcomes directly, CML enables estimation of individual treatment effects (ITE) and aggregation to population-level average treatment effects (ATE), while accommodating complex, high-dimensional covariates [6]. Within this framework, several meta-learning strategies—most commonly the S-Learner, T-Learner, and X-Learner—have been developed, differing in how treatment assignment and outcome models are specified [7].

In parallel, radiomics has been increasingly applied in medical imaging analysis to extract quantitative features that characterize tumor morphology and heterogeneity from routine imaging data [8]. These radiomic features encode underlying pathological information and are frequently associated with both treatment selection and patient outcomes. Consequently, in treatment effect evaluation, radiomic features can act as high-dimensional confounders that require appropriate adjustment. Traditional causal inference methods often struggle to accommodate such complex feature spaces, whereas causal machine learning frameworks can incorporate radiomic and clinical variables jointly to model potential outcomes. This integration enables estimation of treatment effects at both the population and individual levels through counterfactual inference [9].

Recent studies have demonstrated the potential of radiomics and machine learning for non-invasive tumor characterization, including preoperative grade assessment, treatment-response prediction, and survival modeling across different malignancies [10–14]. These studies support the broader methodological relevance of imaging-derived quantitative features in precision oncology. However, performance estimates reported in other tumor types should not be directly extrapolated to osteosarcoma or to causal treatment-effect estimation. In osteosarcoma, prior radiomics studies have mainly focused on prognostic prediction, chemotherapy-response assessment, and imaging-based risk stratification rather than causal estimation of treatment effects [15]. A systematic review of osteosarcoma radiomics also highlighted that many existing studies remain limited by small sample sizes, insufficient external validation, and heterogeneous imaging protocols, underscoring the need for cautious interpretation and transparent methodological reporting [16]. Therefore, integrating radiomic features with causal inference should be regarded as an exploratory methodological extension rather than a replacement for adequately powered clinical validation [17–19].

Osteosarcoma is the most common primary malignant bone tumor in adolescents and young adults, characterized by aggressive biological behavior and a high risk of early metastasis [20]. Despite advances in multimodal treatment, the prognosis remains poor for patients with metastatic or recurrent disease, with reported 5-year survival rates substantially lower than those of localized cases [21].

The current standard of care consists of neoadjuvant chemotherapy (NAC) followed by surgical resection. Multi-agent chemotherapy regimens have improved survival outcomes, and a favorable histopathological response to NAC is associated with better prognosis [22, 23]. However, the optimal number of NAC cycles and the timing of surgery remain uncertain. Prior clinical studies have examined associations among chemotherapy timing, histological response, survival outcomes, and treatment-related toxicity, but the optimal number of preoperative NAC cycles remains uncertain. Existing clinical guidelines do not provide explicit recommendations on chemotherapy cycle duration, and treatment decisions are largely guided by empirical assessment of imaging findings and laboratory indicators [24]. This uncertainty highlights the need for individualized approaches to optimize chemotherapy strategies while balancing potential therapeutic benefit and treatment-related toxicity.

Given the heterogeneity of osteosarcoma and the uncertainty surrounding neoadjuvant chemotherapy cycle intensity, there is a need for exploratory analytical frameworks that can characterize potential treatment-effect variation while explicitly acknowledging the assumptions and limitations of observational data. In this proof-of-concept study, we developed a radiomics-based causal machine learning framework integrating pre-treatment MRI-derived radiomic features and baseline clinical variables. The objectives were to: (1) explore the feasibility of estimating population-level average treatment effects associated with high-cycle versus low-cycle neoadjuvant chemotherapy strategies; (2) generate individualized treatment-effect estimates through counterfactual outcome modeling; and (3) evaluate the interpretability and methodological potential of this framework as a hypothesis-generating tool for future validation studies. The present study was not intended to provide clinical treatment recommendations or to establish definitive causal effects comparable to those from randomized trials. Instead, it aimed to explore whether pre-treatment MRI-derived radiomic features and baseline clinical variables could be integrated into a potential-outcomes-based causal meta-learning workflow for exploratory treatment-effect estimation in a small retrospective osteosarcoma cohort. The resulting estimates were interpreted strictly as hypothesis-generating outputs under explicit causal assumptions [5, 7].

## Methods

### Data

**Fig. 1:**
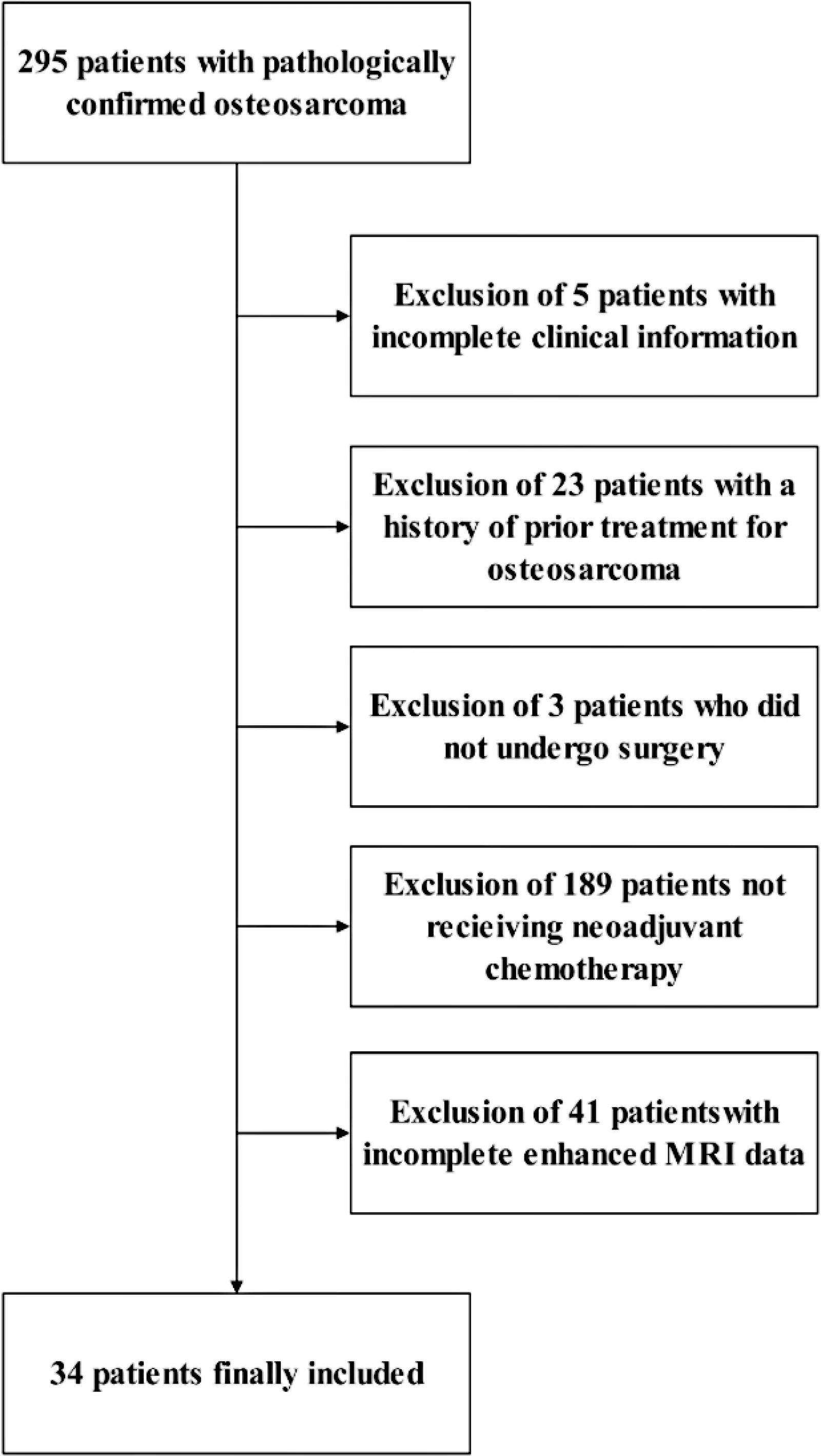
Flowchart illustrating patient selection and cohort construction

This retrospective study was approved by the Ethics Committee of Shanghai Changzheng Hospital (Approval No. 2024SL151), with a waiver of informed consent. A total of 34 patients diagnosed with osteosarcoma between January 2014 and December 2022 were included. Patient selection followed predefined inclusion and exclusion criteria, as summarized in Fig. 1.

Inclusion criteria were as follows: (a) histopathological confirmation of conventional intramedullary osteosarcoma based on biopsy specimens evaluated by an experienced sarcoma pathologist. Patients with other osteosarcoma subtypes, including parosteal osteosarcoma, periosteal osteosarcoma, low-grade central osteosarcoma, telangiectatic osteosarcoma, small-cell osteosarcoma, and high-grade surface osteosarcoma, were not included; (b) receipt of neoadjuvant chemotherapy followed by definitive surgical resection; (c) availability of baseline contrast-enhanced magnetic resonance imaging acquired within 30 days prior to initiation of neoadjuvant chemotherapy, ensuring temporal alignment between imaging features and treatment decision-making; and (d) absence of distant metastasis at initial evaluation.

Exclusion criteria included: (a) prior receipt of targeted therapy before enrollment; (b) history of other primary malignancies; (c) incomplete clinical records or missing key variables required for analysis; and (d) death attributable to causes unrelated to osteosarcoma.

Given the relatively small cohort size, all eligible patients meeting the inclusion criteria during the study period were included to maximize data utilization for causal effect estimation.

### Treatment variable and outcome definition

The treatment variable of interest was the number of neoadjuvant chemotherapy (NAC) cycles administered prior to surgical resection. To reflect common clinical decision thresholds and facilitate causal comparison, patients were categorized into a low-cycle group (< 4 cycles) and a high-cycle group (≥ 4 cycles).

In routine clinical practice, the number of NAC cycles is not randomly assigned but is influenced by baseline disease characteristics, tumor burden, and perceived treatment response. Consequently, treatment assignment may be confounded by patient-specific factors that are also associated with prognosis. To address this issue, relevant clinical variables and radiomic features were incorporated as covariates in the causal machine learning framework to adjust for observed confounding and enable estimation of counterfactual outcomes.

The primary outcome was overall survival (OS), defined as the time from diagnosis to death from any cause. For causal effect estimation, OS was dichotomized based on whether survival exceeded two years, a clinically meaningful time point in osteosarcoma management that is commonly used in outcome stratification. This dichotomized outcome definition was adopted to enable implementation of the binary-outcome causal meta-learning framework in the present small proof-of-concept dataset. The 2-year threshold was selected as a clinically interpretable landmark time point in osteosarcoma outcome assessment. Although this landmark dichotomization inevitably reduces statistical power compared with time-to-event survival modeling, it was adopted as a pragmatic simplification to enable implementation of the binary-outcome causal meta-learning framework in this small proof-of-concept dataset.

Patients with unavailable survival status at the 2-year landmark were treated as having missing outcome information for the binary survival analysis. Given the limited sample size, no formal multiple-imputation procedure was performed. Instead, the extent of missing outcome data was reported descriptively, and the potential influence of missing outcome information was considered in the interpretation of treatment-effect estimates.

### Radiomic feature extraction and feature selection

MRI examinations were performed using a Siemens 1.5-T scanner platform at our institution under a standardized routine musculoskeletal imaging protocol. Pre-treatment MRI acquired within 30 days before neoadjuvant chemotherapy was used for analysis. T1-weighted images acquired with conventional spin-echo sequences were selected for radiomic feature extraction because this sequence was consistently available across all included patients and provided stable anatomical delineation of tumor boundaries. T2-weighted or fat-suppressed T2-weighted images were used as anatomical references during ROI delineation to improve boundary identification but were not included for feature extraction in the present proof-of-concept analysis.

Because this was a retrospective study covering a 9-year recruitment period, detailed scanner model and complete sequence-parameter information could not be uniformly retrieved for all patients. Therefore, no post-acquisition harmonization procedure was applied. Although the use of a single-center Siemens 1.5-T scanner platform and routine institutional protocol may have partially reduced inter-scanner variability, scanner software updates and minor protocol variation over time could not be fully excluded. This issue was considered when interpreting the radiomics-based estimates.

Radiomic feature extraction was performed using pre-treatment T1-weighted magnetic resonance imaging. Regions of interest were manually delineated on T1-weighted images using ITK-SNAP, with reference to T2-weighted or fat-suppressed T2-weighted sequences to ensure accurate tumor boundary identification. Delineation criteria included low signal intensity on T1-weighted images, corresponding high signal intensity on T2-weighted sequences, and consistency with anatomical tumor margins.

Because this retrospective proof-of-concept study was based on a small historical cohort, all ROIs were delineated by a single reader, and formal interobserver or intraobserver reproducibility analysis was not performed. Therefore, intraclass correlation coefficients for ROI delineation were not available. This limitation was explicitly considered when interpreting the robustness of radiomic features and downstream causal treatment-effect estimates. Following segmentation, radiomic features were extracted using the open-source PyRadiomics library. The extracted feature set included first-order statistical features, shape-based descriptors, texture features, and wavelet-transformed features. To reduce dimensionality and mitigate overfitting in subsequent causal modeling, feature selection was conducted using Least Absolute Shrinkage and Selection Operator (LASSO) regression with ten-fold cross-validation. Radiomic features with non-zero coefficients were retained and integrated with clinical variables as covariates in the causal machine learning framework.

### Causal machine learning framework and treatment effect estimation

To estimate causal effects associated with different neoadjuvant chemotherapy cycle strategies, we implemented three widely used causal meta-learning approaches: the S-Learner, T-Learner, and X-Learner. All models were trained using a shared set of covariates, including baseline clinical variables and selected radiomic features.

Given the limited sample size, model training was implemented using the AutoGluon framework to provide a standardized exploratory modeling workflow with reduced dependence on manual hyperparameter tuning. However, we do not claim that ensemble learning can overcome the statistical limitations imposed by the small sample size. In particular, the use of flexible machine learning models in this cohort remains vulnerable to overfitting, model instability, and sampling variability. Therefore, AutoGluon was used only as a technical implementation platform for proof-of-concept counterfactual modeling, and the resulting ATE and ITE estimates were interpreted as exploratory and hypothesis-generating rather than as validated treatment-effect estimates.

Because of the limited sample size, the implemented models were not intended to establish a clinically deployable prediction model or to provide definitive evidence of treatment-effect heterogeneity. Instead, the modeling strategy was used to demonstrate the technical feasibility of integrating radiomic covariates into a causal meta-learning workflow. Model-derived ATE and ITE estimates were therefore interpreted as exploratory, hypothesis-generating quantities. The 8:2 split was used only as an internal technical check of model fitting, and performance metrics derived from the small test set were not considered evidence of generalizable predictive performance.

In the S-Learner approach, treatment assignment was included as an additional input feature alongside covariates, and a single model was trained to predict survival outcomes. In contrast, the T-Learner approach involved training two separate outcome models corresponding to the high-cycle and low-cycle treatment groups. The X-Learner further extended the T-Learner by incorporating propensity score–based weighting to improve robustness of treatment effect estimation, particularly in the presence of imbalanced treatment group sizes.

Let Y(1) and Y(0) denote the potential survival outcomes under high-cycle and low-cycle chemotherapy strategies, respectively. The individual treatment effect (ITE) for a patient with covariates X was defined as the difference in predicted survival probabilities under the two counterfactual scenarios:

$$\:ITE\left(X\right)\hspace{0.17em}=\hspace{0.17em}E\left[Y\right(1\left)\:\right|\:X]\hspace{0.17em}-\hspace{0.17em}E[Y\left(0\right)\:\left|\:X\right]$$

The average treatment effect (ATE) was calculated as the mean of individual treatment effects across the study population.

For the X-Learner, propensity scores—defined as the probability of receiving the high-cycle treatment conditional on covariates—were estimated using logistic regression. These propensity scores were then used to weight counterfactual outcome predictions, yielding stabilized estimates of individual treatment effects.

**Fig. 2:**
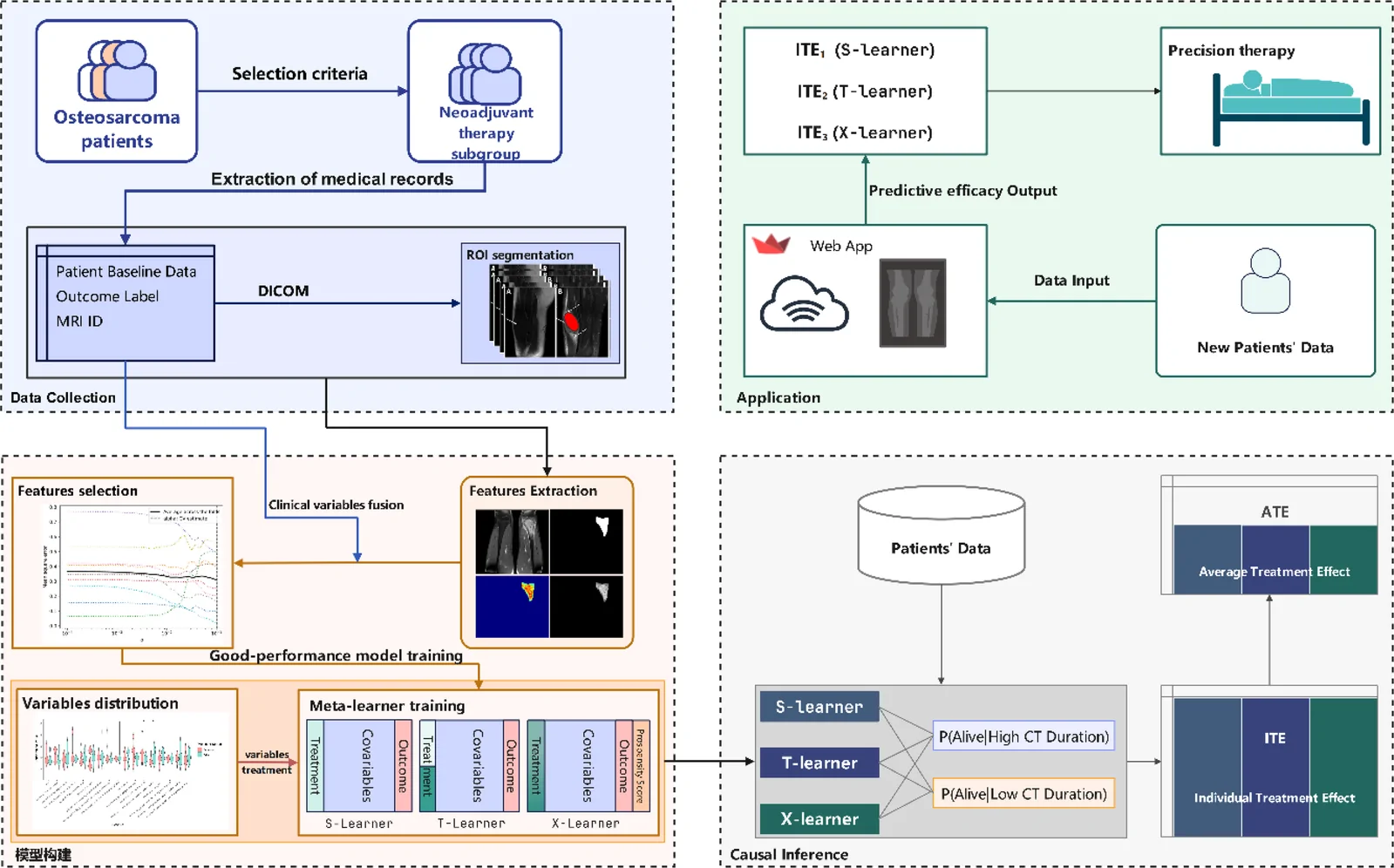
Overview of the radiomics-based causal machine learning workflow for osteosarcoma

To quantify uncertainty in ATE estimation, a nonparametric bootstrap procedure with 500 resampling iterations was applied. The standard error and corresponding 95% confidence interval of the ATE were derived from the bootstrap distribution. This causal framework relies on standard assumptions of consistency, conditional exchangeability, and positivity. These assumptions are not fully testable in retrospective observational data. Therefore, estimated ATE and ITE values should be interpreted as conditional estimates under the observed covariates and specified models, rather than as definitive causal effects. The overall workflow of the proposed radiomics-based causal machine learning framework is illustrated in Fig. 2.

To improve the interpretability of individualized treatment-effect estimates, illustrative patient-level examples were summarized. These examples were used only to demonstrate how ITE outputs can be interpreted within the proposed framework and were not intended to provide treatment recommendations.

### Statistical analysis and software

Descriptive and comparative statistical analyses were performed according to variable types. Categorical variables were compared using the chi-square test or Fisher’s exact test, as appropriate. Continuous variables were analyzed using the Wilcoxon rank-sum test. All statistical tests were two-sided, and a p-value < 0.05 was considered statistically significant.

Statistical analyses were primarily conducted using R (version 4.1.3). Visualization and summary analyses were performed with commonly used R packages, including gtsummary, ggplot2, ggpubr, and tidyverse. Radiomic feature extraction was implemented in Python using the PyRadiomics library. Feature selection and causal model training were conducted in Python using scikit-learn and AutoGluon, respectively.

To ensure reproducibility and analytical reliability, all analysis scripts were version-controlled, and computational environments were managed using conda to isolate dependencies. Key analytical steps were independently verified to minimize implementation errors.

## Results

### Patient recruitment and characteristics

A total of 34 patients with osteosarcoma who met the predefined inclusion and exclusion criteria were included in the analysis. The median age of the cohort was 21 years (interquartile range [IQR], 14–45 years), with 24 males (70.6%) and 10 females (29.4%). Patients were categorized according to the number of neoadjuvant chemotherapy cycles into a high-cycle group (≥ 4 cycles, *n* = 16) and a low-cycle group (< 4 cycles, *n* = 18). A significant imbalance in sex distribution was observed between the high-cycle and low-cycle groups, indicating potential baseline confounding that should be considered when interpreting subsequent treatment-effect estimates.

Baseline demographic and clinical characteristics of the study population are summarized in Table 1. Overall, the distributions of age, Ki-67 expression grade, progression-free survival, and overall survival were comparable between the two chemotherapy cycle groups. As expected, the number of chemotherapy cycles differed significantly between groups (*p* < 0.001), reflecting the predefined treatment categorization. Overall survival information was unavailable for 7 patients, including 4 patients in the high-cycle group and 3 patients in the low-cycle group. The presence of missing outcome information was considered when interpreting the subsequent causal effect estimates.

**Table 1:** Characteristics of patients included group by chemotherapy cycle

Characteristic	Overall 1 *N* = 34	High cycle 1 *N* = 16	Low cycle 1 *N* = 18	*p*-value 2
Gender				0.016
Male	24 / 34 (71%)	15 / 16 (94%)	9 / 18 (50%)	
Female	10 / 34 (29%)	1 / 16 (6.3%)	9 / 18 (50%)	
Age	22 (14–45)	23 (14–55)	20 (14–31)	0.5
Ki-67 Grade				0.11
1	8 / 34 (24%)	3 / 16 (19%)	5 / 18 (28%)	
2	11 / 34 (32%)	8 / 16 (50%)	3 / 18 (17%)	
3	15 / 34 (44%)	5 / 16 (31%)	10 / 18 (56%)	
Chemotherapy Duration	3.5 (3.0–4.5)	4.8 (4.0–5.0)	3.0 (3.0–3.0)	< 0.001
Progression-Free Survival	1.1 (1.0–2.6)	1.0 (1.0–2.0)	2.0 (1.0–3.0)	0.2
Unknown	6	4	2	
Overall Survival	2.0 (1.0–3.0)	2.0 (1.0–2.0)	2.0 (1.0–3.0)	0.6
Unknown	7	4	3	

1 n / N (%); Median (Q1-Q3)

2 Pearson’s Chi-squared test; one-way analysis of means

Baseline characteristics stratified by sex are presented in Table 2. No substantial differences were observed in age distribution, Ki-67 grade, or overall survival between male and female patients. A difference in progression-free survival was observed between sex groups (*p* = 0.034).

**Table 2:** Characteristics of patients included group by Gender

Characteristic	Overall 1 *N* = 34	Male 1 *N* = 24	Female 1 *N* = 10	*p*-value 2
Age	22 (14–45)	22 (15–54)	19 (12–31)	0.3
Ki-67 Grade				0.6
1	8 / 34 (24%)	5 / 24 (21%)	3 / 10 (30%)	
2	11 / 34 (32%)	9 / 24 (38%)	2 / 10 (20%)	
3	15 / 34 (44%)	10 / 24 (42%)	5 / 10 (50%)	
Chemotherapy Duration	3.5 (3.0–4.5)	4.0 (3.0–5.0)	3.0 (3.0–3.0)	0.054
Progression-Free Survival	1.1 (1.0–2.6)	1.0 (1.0–2.0)	3.0 (1.1–3.0)	0.034
Unknown	6	5	1	
Overall Survival	2.0 (1.0–3.0)	2.0 (1.0–2.0)	3.0 (1.5–3.5)	0.068
Unknown	7	5	2	

1 Median (Q1 - Q3); n / N (%)

2 One-way analysis of means; Pearson’s Chi-squared test

### Radiomic feature extraction and selection

Radiomic features were extracted from the manually delineated regions of interest using the PyRadiomics library. Because formal interobserver or intraobserver reproducibility assessment was not performed, the stability of segmentation-dependent radiomic features could not be quantified using ICCs in the present study. After LASSO regression with ten-fold cross-validation, 14 radiomic features with non-zero coefficients were retained and incorporated together with clinical variables into the causal machine learning framework. A total set of high-dimensional features encompassing first-order statistics, shape descriptors, texture features, and wavelet-transformed features was initially obtained for each patient.

To reduce dimensionality and mitigate the risk of overfitting, feature selection was performed using Least Absolute Shrinkage and Selection Operator (LASSO) regression with ten-fold cross-validation. This procedure resulted in the retention of 14 radiomic features with non-zero coefficients, which were subsequently incorporated together with clinical variables into the causal machine learning models.

**Fig. 3:**
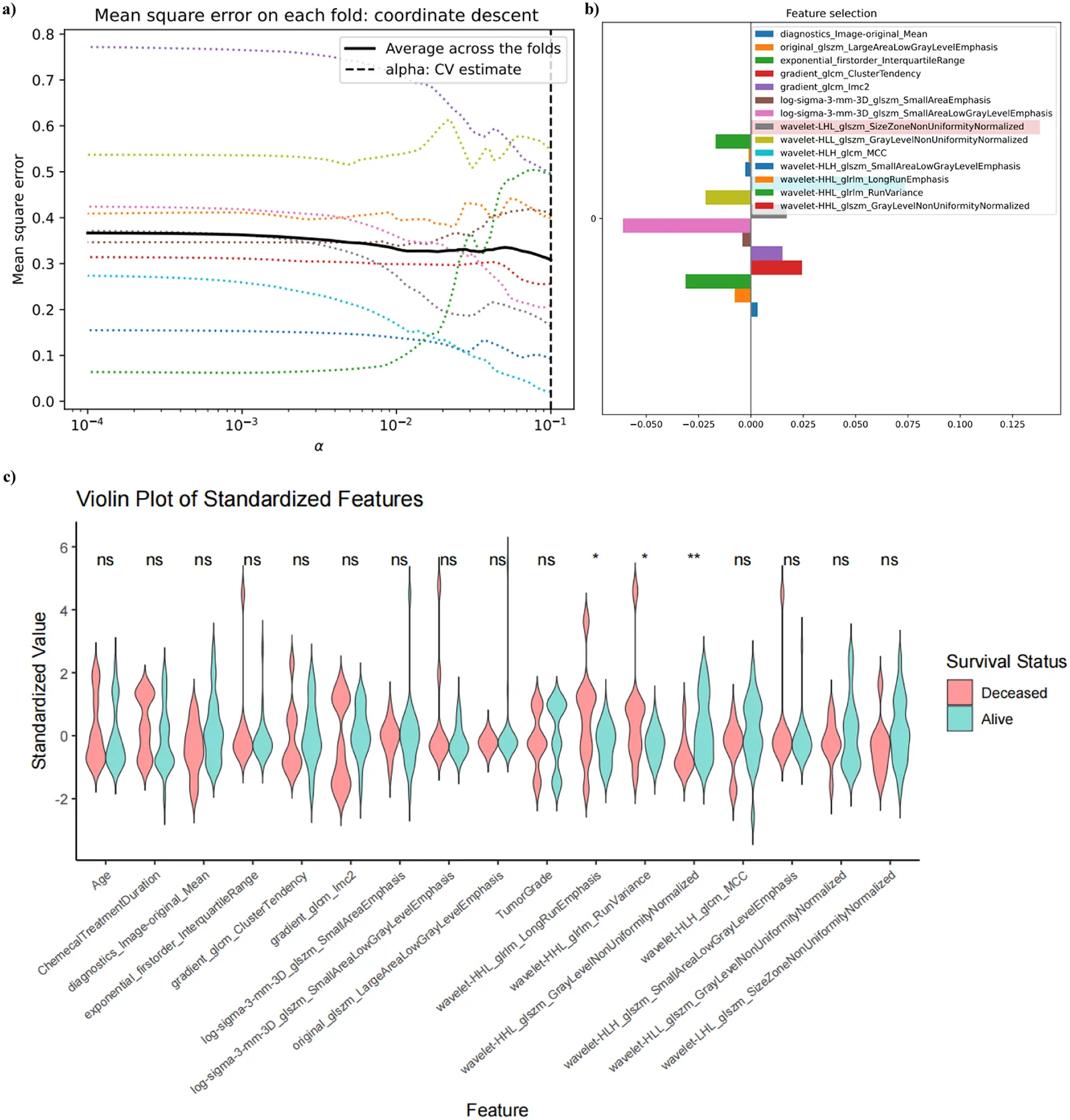

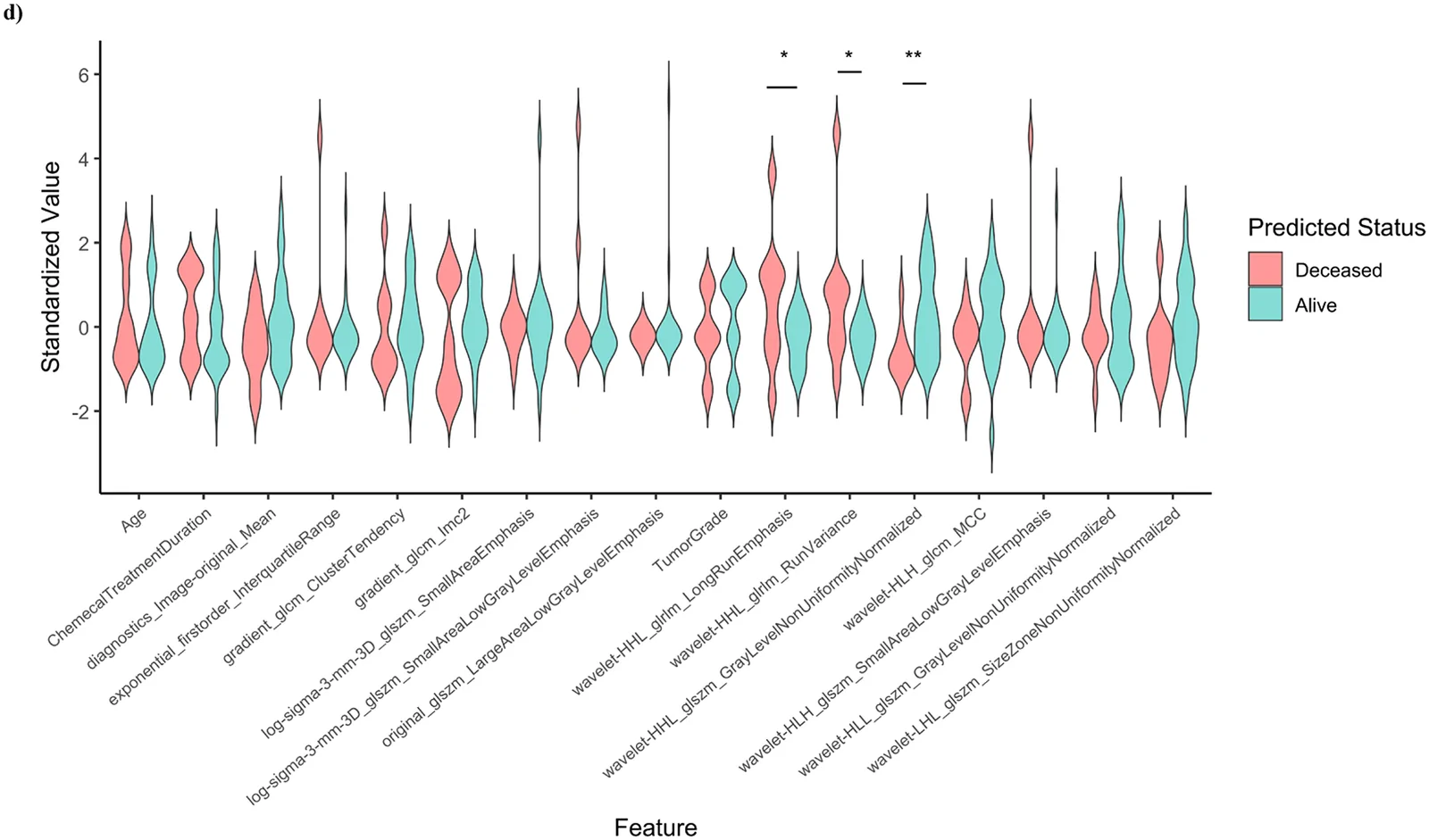
Radiomic feature selection and integration with clinical variables. (**a**) Ten-fold cross-validated LASSO regression screening of imaging histology features. (**b**) LASSO regression screening for 14 imaging histologic features with non-zero weights. (**c**) Distribution of fusion variables standardized for different chemotherapy duration groups. (**d**) Distribution of fusion variables standardized for different survival outcomes. (*p < 0.05, **p < 0.01)

The distributions of the selected radiomic and clinical features were compared across chemotherapy cycle groups and survival outcome groups. For continuous variables, group-wise differences were assessed using the Wilcoxon rank-sum test. Several radiomic features demonstrated statistically significant differences between groups. Specifically, exponential first-order interquartile range and original GLSZM large area low gray level emphasis differed between high- and low-cycle chemotherapy groups (*p* = 0.046 and *p* = 0.007, respectively). In comparisons stratified by survival outcome, significant differences were observed for wavelet-based GLRLM long run emphasis (*p* = 0.046), GLRLM run variance (*p* = 0.038), and GLSZM gray level non-uniformity normalized (*p* = 0.007). The process of radiomic feature selection and the distribution of selected variables are shown in Fig. 3.

### Predictive modeling and estimation of causal effects

The integrated feature matrix was randomly divided into training and testing sets using an 8:2 ratio. Predictive models required for causal effect estimation were trained using the AutoGluon framework under the good_quality preset. Three causal meta-learning approaches were implemented, including the S-Learner, T-Learner, and X-Learner.

Model performance metrics on the training and testing sets are summarized in Table 3. The S-Learner achieved a training AUC of 1.000, whereas the component models of the T-Learner showed training AUC values of 0.500. These divergent metrics suggest substantial model instability and possible overfitting or failure of reliable discrimination under the extremely limited sample size. Because the 8:2 split produced only a very small test set, the testing performance metrics should not be interpreted as evidence of model validity, stability, or generalizability. They are reported only as descriptive technical outputs of the exploratory modeling process and were not used for model selection.

**Table 3:** Meta-learner trained evaluation

Model	Performance on Training Set				Performance on Testing Set			
	AUC	F1	Accuracy	Precision	AUC	F1	Accuracy	Precision
S-Learner	1.000	0.815	0.688	0.688	1.000	0.833	0.714	0.714
T-Learner (Low-cycle model)	0.500	0.800	0.667	0.667	0.500	0.933	0.875	0.875
T-Learner (High-cycle model)	0.500	0.750	0.600	0.750	1.000	1.000	1.000	1.000

Note: Performance metrics are reported only as descriptive technical outputs of model fitting. Given the extremely small training and testing sets, these metrics should not be interpreted as evidence of model stability, validity, or generalizability

Based on the fitted outcome models, counterfactual survival probabilities under high- and low-cycle chemotherapy strategies were estimated for each patient. Individual treatment effects (ITE) were calculated as the difference between predicted potential outcomes, and average treatment effects (ATE) were obtained by aggregating individual-level estimates.

To illustrate the interpretation of model-derived individualized treatment-effect estimates, two representative patient-level examples with positive and negative ITE values are presented in Table 4. These examples were included solely to demonstrate the output format and interpretability of the proposed framework. Given the limited sample size, treatment-group imbalance, missing outcome information, and instability of the estimated effects, these examples should not be interpreted as evidence for individualized chemotherapy cycle selection or as clinical treatment recommendations.

**Table 4:** Illustrative examples of individualized treatment-effect estimates generated by the proposed framework

Case	Age	Sex	Tumor site	Actual NAC cycle group	Predicted *P*(Y = 1) under high-cycle	Predicted *P*(Y = 1) under low-cycle	ITE	Model-favored strategy for interpretability only	Observed 2-year survival status	Interpretation
1	16	Male	Distal femur	High-cycle	0.72	0.58	0.14	High-cycle	Survived	Higher model-estimated survival probability under the high-cycle strategy; illustrative positive ITE.
2	21	Female	Proximal tibia	Low-cycle	0.46	0.63	-0.17	Low-cycle	Survived	Higher model-estimated survival probability under the low-cycle strategy; illustrative negative ITE.

NAC, neoadjuvant chemotherapy; ITE, individual treatment effect. Y = 1 indicates survival beyond the 2-year landmark. ITE was calculated as the predicted survival probability under the high-cycle strategy minus the predicted survival probability under the low-cycle strategy. The cases shown are illustrative examples intended only to demonstrate how model-derived ITE outputs may be interpreted within the proposed framework. They should not be considered treatment recommendations or evidence supporting individualized chemotherapy cycle selection

The estimated ATE values and corresponding 95% confidence intervals for the three meta-learning approaches are presented in Table 5. The direction and magnitude of the estimated ATEs differed across learners, with the S-Learner and X-Learner yielding negative estimates (-0.081 and − 0.107, respectively) and the T-Learner yielding a small positive estimate (0.020). The 95% confidence intervals for both the S-Learner and X-Learner crossed zero, whereas the T-Learner interval narrowly excluded zero. These findings indicate that treatment-effect estimates were unstable in this small cohort and should not be interpreted as definitive evidence of benefit or harm associated with high-cycle neoadjuvant chemotherapy. Rather, the results demonstrate that the proposed framework can generate population-level and individualized counterfactual estimates for exploratory analysis.

**Table 5:** Estimated average treatment effects (ATE) of high-cycle versus low-cycle neoadjuvant chemotherapy on survival outcome

Model	ATE (probability difference)	95% Confidence interval	
S-learner	-0.081	(-0.164,0.004)	
T-learner	0.020	(0.008,0.030)	
X-learner	-0.107	(-0.191,0.011)	

Note: ATE represents the difference in predicted survival probability between high-cycle and low-cycle chemotherapy strategies

## Discussion

Traditional machine learning models are commonly used in medical research to predict clinical outcomes based on observed patient characteristics. Although such models can achieve favorable predictive performance, their outputs are typically limited to outcome probabilities under observed treatment conditions and do not directly address causal questions regarding treatment effects. As a result, their ability to evaluate alternative treatment strategies in observational settings is constrained.

In contrast, causal machine learning aims to model potential outcomes under alternative treatment strategies, enabling estimation of both population-level average treatment effects and individual-level treatment effects. By framing treatment evaluation in a counterfactual context, this approach provides a methodological basis for comparing expected outcomes under different treatment intensities. In the present study, the causal framework allowed exploratory assessment of neoadjuvant chemotherapy cycle strategies beyond conventional risk prediction, offering a proof-of-concept workflow for hypothesis-generating treatment-effect evaluation.

The main methodological advantage of the proposed framework is that it shifts the analytical target from conventional prognostic prediction to counterfactual treatment-effect estimation [1]. Standard radiomics-based models generally estimate outcome risk or treatment response under observed clinical management, whereas causal meta-learners attempt to estimate potential outcomes under alternative treatment strategies and thereby generate ATE and ITE measures [7]. This distinction is important because response prediction and treatment-effect estimation answer related but different clinical questions [11]. Nevertheless, the validity of causal treatment-effect estimation depends on adequate sample size, reliable covariate measurement, sufficient overlap between treatment groups, and plausible causal assumptions. In small retrospective datasets, individualized effect estimates may be highly sensitive to model specification, treatment-group imbalance, missing outcome information, and unmeasured confounding. Therefore, the present study should be interpreted as a proof-of-concept demonstration of workflow feasibility rather than evidence that radiomics-based causal machine learning can support individualized chemotherapy selection.

In causal analyses based on observational data, individual treatment-effect estimates are inherently subject to uncertainty arising from unmeasured or imperfectly measured confounding. Although causal machine learning enables individualized counterfactual estimation, ITEs should be interpreted as conditional estimates under the assumptions of the specified model and observed covariates. In the present analysis, the S-Learner and X-Learner produced negative estimates, whereas the T-Learner produced a small positive estimate. This directional inconsistency suggests that the estimated treatment effects were sensitive to model specification and sampling variability. In addition, confidence intervals crossed zero for two of the three learners, indicating that no robust population-level treatment effect could be established in this cohort. Therefore, the ATE and ITE estimates should be interpreted as exploratory outputs of a methodological framework rather than evidence supporting a specific chemotherapy cycle strategy.

The practical implementation of causal inference in this cohort remains vulnerable to unmeasured confounding [11, 24]. In routine clinical practice, the number of neoadjuvant chemotherapy cycles may be influenced by early radiological response, histopathological tumor necrosis rate, treatment-related toxicity, physician preference, and patient preference. Because these variables were not fully recorded or incorporated into the present analysis, they may have influenced both treatment-cycle assignment and survival outcomes. For example, patients with more aggressive disease or poor early response may have been more likely to receive extended chemotherapy, which could bias the estimated effect of high-cycle treatment toward worse outcomes. Conversely, patients who were able to tolerate additional chemotherapy may have had better baseline physiological reserve, potentially biasing estimates toward a more favorable effect.

Accordingly, the present analysis should not be interpreted as approximating the evidentiary strength of a randomized trial. Rather, it represents an exploratory application of a potential-outcomes framework to historical observational data, with all causal interpretations remaining conditional on unverifiable assumptions and the limited covariates available in this cohort.

Sensitivity analysis frameworks, including approaches based on counterfactual explanations or quantification of unmeasured confounding, have been proposed to assess the robustness of causal conclusions. Such methods were not explicitly implemented in the present study. Future studies with larger cohorts should incorporate formal sensitivity analyses to evaluate how violations of causal assumptions may influence average and individualized treatment-effect estimates.

Radiomic features provide quantitative representations of tumor morphology and heterogeneity derived from routine medical imaging. From a causal perspective, such features may influence both treatment decision-making and patient outcomes, thereby acting as potential confounders in observational studies of treatment effects. Explicitly incorporating radiomic features into causal machine learning models allows these high-dimensional imaging-derived variables to be considered within a unified analytical framework. In the present study, integrating radiomic and clinical features enabled exploratory estimation of counterfactual outcomes under alternative chemotherapy cycle strategies while accounting for complex baseline heterogeneity.

The performance and reliability of causal machine learning models depend on the quality of input data and the adequacy of feature representation. In this study, radiomic feature extraction and region-of-interest delineation were performed following standardized procedures; nevertheless, variations in image acquisition and segmentation accuracy may influence downstream model estimates. Such variability is an inherent consideration in radiomics-based analyses and underscores the importance of consistent imaging protocols. Given the small sample size, the implemented machine learning models were used only to demonstrate the technical feasibility of integrating radiomic covariates into a causal meta-learning workflow. The use of ensemble-based modeling should not be interpreted as overcoming the limitations of the small cohort. Model performance metrics were secondary descriptive outputs and do not establish predictive validity, model stability, or generalizability.

In addition to unmeasured clinical variables, baseline imbalance and temporal heterogeneity may have further affected the reliability of the exploratory estimates [16]. The high-cycle treatment group included a substantially higher proportion of male patients than the low-cycle group (94% vs. 50%, *p* = 0.016). Although sex was included as an observed covariate, adjustment for this imbalance is statistically unstable in a 34-patient cohort and may not fully address residual confounding or limited covariate overlap. Furthermore, the 9-year recruitment period from 2014 to 2022 may have introduced temporal heterogeneity related to treatment practice, imaging acquisition, and clinical management. These factors may further complicate the interpretation of counterfactual treatment-effect estimates derived from this retrospective dataset.

Overall, this study demonstrates the feasibility of integrating radiomic features with causal meta-learning strategies to generate counterfactual treatment-effect estimates in a small retrospective osteosarcoma cohort. However, the inconsistent ATE estimates across learners, limited sample size, treatment-group imbalance, missing outcome information, and potential unmeasured confounding preclude any definitive conclusion regarding the superiority or inferiority of high-cycle versus low-cycle neoadjuvant chemotherapy. The findings should therefore be interpreted as hypothesis-generating and methodological rather than clinically actionable.

### Limitations

Several limitations of this study should be acknowledged. First, this was a retrospective single-center study with a relatively small sample size. Although all eligible patients within the study period were included to maximize data utilization, the cohort size was insufficient to support definitive causal inference or reliable assessment of treatment-effect heterogeneity. Therefore, the present study should be regarded as a methodological feasibility and proof-of-concept investigation rather than a clinically validated treatment-effect model. Crucially, results from this study cannot be interpreted as valid clinical evidence regarding neoadjuvant chemotherapy cycle intensity or used for clinical recommendations. Accordingly, the observed patterns may be strongly influenced by sampling variability, model instability, and residual confounding, and should be treated strictly as exploratory.

Second, the estimated treatment effects were unstable across causal meta-learners. The inconsistency in ATE direction across learners further underscores the instability of treatment-effect estimation in this dataset. This instability likely reflects the combined effects of limited sample size, treatment-group imbalance, missing outcome information, and model dependence. Consequently, no reliable conclusion regarding the superiority or inferiority of high-cycle versus low-cycle neoadjuvant chemotherapy can be drawn from the present analysis.

Third, follow-up duration was limited for a proportion of patients, and right-censoring is an inherent challenge in survival analysis. To facilitate implementation of the proposed binary-outcome causal meta-learning framework, overall survival was dichotomized at a 2-year landmark time point. Although this threshold is clinically interpretable, dichotomization inevitably results in loss of temporal information and may further reduce statistical power in a small cohort. Future studies incorporating longer follow-up and time-to-event causal modeling approaches, such as causal survival forests or counterfactual survival models, may provide more informative treatment-effect estimates.

Fourth, missing outcome information may have further affected the reliability of the estimated treatment effects. Overall survival status at the 2-year landmark was unavailable for 7 patients, representing approximately one-fifth of the cohort. Because the sample size was limited, formal multiple imputation or robust missing-data sensitivity analysis was not performed. Therefore, the estimated ATE and ITE values may be sensitive to assumptions regarding the outcomes of these patients.

Fifth, baseline imbalance between treatment-cycle groups represents an important source of uncertainty. In particular, sex distribution differed significantly between the high-cycle and low-cycle groups. Although sex and other available clinical variables were incorporated as covariates in the causal machine learning framework, the small sample size limited the ability to fully adjust for baseline imbalance or to verify positivity across covariate strata. Therefore, residual confounding related to observed baseline differences may have influenced the estimated treatment effects.

Sixth, residual and unmeasured confounding cannot be excluded. The number of neoadjuvant chemotherapy cycles may have been influenced by factors that were not fully captured in the available dataset, including histological response, tumor necrosis rate, early clinical response, treatment-related toxicity, physician preference, patient preference, and changes in clinical management over time. Some of these factors may be associated with both treatment-cycle assignment and survival outcomes. For example, patients with poor early response or more aggressive disease may have been more likely to receive extended chemotherapy, which could bias the estimated effect of high-cycle treatment toward worse outcomes. Conversely, patients able to tolerate additional chemotherapy may have had better baseline health status, potentially biasing estimates toward a more favorable effect. Therefore, the estimated ATE and ITE values should be interpreted as conditional exploratory estimates under observed covariates rather than definitive causal effects.

Seventh, radiomics-related limitations should be considered. Radiomic features were extracted only from T1-weighted images. Although T2-weighted and fat-suppressed T2-weighted sequences were used as anatomical references for ROI delineation, multiparametric MRI sequence-such as diffusion-weighted imaging (DWI), apparent diffusion coefficient (ADC) maps, and contrast-enhanced sequences were not incorporated. These advanced parameters provide highly complementary functional data regarding tumor cellularity, vascularity, and heterogeneity, which might contribute substantial additional predictive and stratification value to counterfactual inference. The exclusion of these imaging biomarkers represents a limitation, and future prospective studies should evaluate whether multi-sequence radiomics can more comprehensively capture osteosarcoma heterogeneity. Another important limitation concerns the reproducibility of manual ROI delineation. In the present retrospective study, tumor ROIs were delineated by a single experienced reader, and formal interobserver or intraobserver ICC analysis was not performed. Therefore, the potential influence of segmentation variability on radiomic feature values could not be quantitatively assessed. Because radiomic features are sensitive to ROI definition, segmentation-related measurement error may have influenced feature selection and downstream causal treatment-effect estimates. Future studies should include repeated or multi-reader segmentation, report ICC values for radiomic features, and retain only reproducible features before causal modeling. Future studies should evaluate multiparametric MRI-based radiomics and apply standardized acquisition or harmonization strategies to improve reproducibility across scanners and institutions. Although all images were acquired using a single-center Siemens 1.5-T scanner platform, detailed scanner model and complete sequence-parameter information were not uniformly available because of the retrospective design and long recruitment period. Scanner software updates and minor protocol variation over time may therefore have influenced radiomic feature extraction and downstream treatment-effect estimation.

Eighth, treatment strategies for osteosarcoma remain an area of active clinical investigation, including ongoing discussion regarding the sequencing of chemotherapy and surgery. The present study focused specifically on evaluating differences in neoadjuvant chemotherapy cycle intensity and did not aim to compare chemotherapy-first and surgery-first strategies. Consequently, the findings should be interpreted within the scope of this treatment context.

Finally, this study included only patients with conventional intramedullary osteosarcoma. Therefore, the findings may not be generalizable to other histological subtypes of osteosarcoma, such as parosteal, periosteal, low-grade central, telangiectatic, small-cell, or high-grade surface osteosarcoma. Despite these limitations, the study demonstrates the feasibility of integrating radiomics with causal machine learning to generate exploratory individualized treatment-effect estimates, providing a methodological foundation for future multicenter and prospective investigations.

## Conclusion

This study demonstrates the methodological feasibility of integrating radiomics with causal machine learning to explore treatment-effect estimation associated with neoadjuvant chemotherapy cycle intensity in osteosarcoma. By modeling counterfactual outcomes, the proposed framework provides an exploratory approach for generating average and individualized treatment-effect estimates from routinely acquired imaging and clinical data. However, given the limited sample size, retrospective single-center design, treatment-group imbalance, missing outcome information, and potential unmeasured confounding, the estimated effects should be interpreted as hypothesis-generating rather than clinically actionable. Future studies should validate this framework in larger, multicenter cohorts with standardized imaging protocols, adequate event counts, longer follow-up, and time-to-event causal modeling before any clinical utility can be assessed.

## Supplementary Information

Below is the link to the electronic supplementary material.

Supplementary Material 1

Supplementary Material 2

## Data Availability

The datasets generated and/or analyzed during the current study are not publicly available due to patient privacy restrictions but are available from the corresponding author on reasonable request.

## Abbreviations

ADC: Apparent diffusion coefficient
ATE: Average treatment effect
AUC: Area under the curve
AUROC: Area under the receiver operating characteristic curve
CML: Causal machine learning
DWI: Diffusion-weighted imaging
EPV: Events-per-variable
GLRLM: Gray-level run length matrix
GLSZM: Gray-level size zone matrix
ICC: Intraclass correlation coefficient
ITE: Individual treatment effect
LASSO: Least Absolute Shrinkage and Selection Operator
MRI: Magnetic resonance imaging
NAC: Neoadjuvant chemotherapy
OS: Overall survival

## References

[1] Pearl J. Causality: models, reasoning, and inference. 2nd ed. 2009.

[2] Angrist JD, Imbens GW, Rubin DB. Identification of Causal Effects Using Instrumental Variables. J Am Stat Assoc. 1996;91:444–55. 10.1080/01621459.1996.10476902.

[3] Austin PC. An Introduction to Propensity Score Methods for Reducing the Effects of Confounding in Observational Studies. Multivar Behav Res. 2011;46:399–424. 10.1080/00273171.2011.568786. PMC3144483 21818162

[4] Rosenbaum PR, Rubin DB. The Central Role of the Propensity Score in Observational Studies for Causal Effects. Biometrika. 1983;70:41–55. 10.2307/2335942.

[5] Feuerriegel S, Frauen D, Melnychuk V, et al. Causal machine learning for predicting treatment outcomes. Nat Med. 2024;30:958–68. 10.1038/s41591-024-02902-1. 38641741

[6] Grimmer J, Messing S, Westwood SJ. Estimating Heterogeneous Treatment Effects and the Effects of Heterogeneous Treatments with Ensemble Methods. Political Anal. 2017;25:413–34. 10.1017/pan.2017.15.

[7] Künzel SR, Sekhon JS, Bickel PJ, et al. Metalearners for estimating heterogeneous treatment effects using machine learning. Proc Natl Acad Sci USA. 2019;116:4156–65. 10.1073/pnas.1804597116. 30770453 PMC6410831

[8] Guo Y, Li T, Gong B, et al. From Images to Genes: Radiogenomics Based on Artificial Intelligence to Achieve Non-Invasive Precision Medicine in Cancer Patients. Adv Sci. 2025;12:2408069. 10.1002/advs.202408069. PMC11727298 39535476

[9] Ghosh D, Mastej E, Jain R, et al. Causal inference in radiomics: framework, mechanisms, and algorithms. Front Neurosci. 2022;16. 10.3389/fnins.2022.884708. PMC9261933 35812228

[10] Chen H, Zhang X, Wang X, et al. MRI-based radiomics signature for pretreatment prediction of pathological response to neoadjuvant chemotherapy in osteosarcoma: a multicenter study. Eur Radiol. 2021;31:7913–24. 10.1007/s00330-021-07748-6. 33825032

[11] Harun N, Sparapani R, Laud PW, et al. Treatment Effect Estimation With Potential Outcomes for a Single-Arm Trial Compared With Historical Controls: A Case Study of Locally Recurrent Head and Neck Squamous Cell Carcinoma Patients Treated With Adjuvant Nivolumab. Head Neck. 2025;47:2990–7. 10.1002/hed.28215. 40525328

[12] Benfante V, Salvaggio G, Ali M, et al. Grading and Staging of Bladder Tumors Using Radiomics Analysis in Magnetic Resonance Imaging. In: Foresti GL, Fusiello A, Hancock E, editors. Image Analysis and Processing - ICIAP 2023 Workshops. Cham: Springer Nature Switzerland; 2024. pp. 93–103. 10.1007/978-3-031-51026-7_9.

[13] Canfora I, Cutaia G, Marcianò M, et al. A Predictive System to Classify Preoperative Grading of Rectal Cancer Using Radiomics Features. In: Mazzeo PL, Frontoni E, Sclaroff S, et al. editors. Image Analysis and Processing. ICIAP 2022 Workshops. Cham: Springer International Publishing; 2022. pp. 431–40. 10.1007/978-3-031-13321-3_38.

[14] Chirra M, Elliott A, Shaikh H, et al. Molecular Profiling and Real-world Outcomes of BRAF V600E–Mutated Papillary Thyroid Cancer. Clin Cancer Res. 2026;32:1110–9. 10.1158/1078-0432.CCR-25-3932. 41543354

[15] Zheng F, Yin P, Liang K, et al. Fusion Radiomics-Based Prediction of Response to Neoadjuvant Chemotherapy for Osteosarcoma. Acad Radiol. 2024;31:2444–55. 10.1016/j.acra.2023.12.015. 38151381

[16] Zhong J, Hu Y, Si L, et al. A systematic review of radiomics in osteosarcoma: utilizing radiomics quality score as a tool promoting clinical translation. Eur Radiol. 2021;31:1526–35. 10.1007/s00330-020-07221-w. 32876837

[17] Ye R, Yuan Q, You W, et al. Identification of the shared gene signatures in retinoblastoma and osteosarcoma by machine learning. Sci Rep. 2024;14:31355. 10.1038/s41598-024-82789-7. 39733097 PMC11682156

[18] Yuan Q, Hong Z, Ye R, et al. Integrating Deep Feature Extraction and MRI Radiomics for Survival Prediction in Breast Cancer After Neoadjuvant Chemotherapy. Acad Radiol. 2026;33:872–88. 10.1016/j.acra.2025.10.050. 41253608

[19] Yuan Q, Ye R, Qian Y, et al. Machine learning and SHAP value interpretation for predicting the response to neoadjuvant chemotherapy and long-term clinical outcomes in Chinese female breast cancer. Ann Med. 2025;57:2541316. 10.1080/07853890.2025.2541316. 40754951 PMC12322993

[20] Meltzer PS, Helman LJ. New Horizons in the Treatment of Osteosarcoma. N Engl J Med. 2021;385:2066–76. 10.1056/NEJMra2103423. 34818481

[21] Gorlick R, Janeway K, Lessnick S, et al. Children’s Oncology Group’s 2013 Blueprint for Research: Bone Tumors. Pediatr Blood Cancer. 2013;60:1009–15. 10.1002/pbc.24429. 23255238 PMC4610028

[22] Biermann JS, Hirbe A, Ahlawat S, et al. Bone cancer, version 2.2025, NCCN clinical practice guidelines in oncology. J Natl Compr Canc Netw. 2025;23. 10.6004/jnccn.2025.0017. 40203873

[23] Meyers PA, Schwartz CL, Krailo MD, et al. Osteosarcoma: the addition of muramyl tripeptide to chemotherapy improves overall survival–a report from the Children’s Oncology Group. J Clin Oncol. 2008;26:633–8. 10.1200/JCO.2008.14.0095. 18235123

[24] Danese MD, Groundland JS. Effect of chemotherapy and surgery timing on mortality in upper and lower extremity osteosarcoma. JNCI: J Natl Cancer Inst. 2025;117:611–8. 10.1093/jnci/djae229. 39302698

