# Supplementary material for: Radiomics-based causal machine learning for exploratory treatment-effect estimation of neoadjuvant chemotherapy cycle intensity in osteosarcoma: a proof-of-concept study
Source: BMC Med Imaging. 2026 Jun 30;26:467. doi: 10.1186/s12880-026-02533-7 (PMC13599299; doi:10.1186/s12880-026-02533-7)
Supplement: Supplementary file 1 [file 12880_2026_2533_MOESM1_ESM.docx]

**STROBE Statement—Checklist of items that should be included in reports of observational studies**

Manuscript: Radiomics-Based Causal Machine Learning for Exploratory Treatment-Effect Estimation of Neoadjuvant Chemotherapy Cycle Intensity in Osteosarcoma: A Proof-of-Concept Study

| **Section** | **Item No.** | **Recommendation** | **Page / section where reported** | **Relevant text from manuscript** |
| --- | --- | --- | --- | --- |
| Title and abstract | 1a | Indicate the study design with a commonly used term in the title or the abstract. | Title; Abstract | The title identifies the study as a proof-of-concept study. The abstract states that this is a retrospective single-center study including 34 patients. |
| Title and abstract | 1b | Provide in the abstract an informative and balanced summary of what was done and what was found. | Abstract | The abstract summarizes the background, objective, methods, main exploratory findings, and cautious conclusion, including instability of ATE estimates and non-actionable interpretation. |
| Introduction: Background/rationale | 2 | Explain the scientific background and rationale for the investigation being reported. | Section 1, Background | The Background explains causal inference, radiomics, osteosarcoma treatment context, uncertainty regarding NAC cycle intensity, and rationale for exploratory treatment-effect estimation. |
| Introduction: Objectives | 3 | State specific objectives, including any prespecified hypotheses. | Section 1, final paragraph | Objectives are listed: explore feasibility of estimating ATE, generate ITE estimates through counterfactual outcome modeling, and evaluate the interpretability and methodological potential of the framework. |
| Methods: Study design | 4 | Present key elements of study design early in the paper. | Sections 2.1 and 2.2 | The study is described as a retrospective single-center cohort and proof-of-concept causal machine learning framework. |
| Methods: Setting | 5 | Describe the setting, locations, and relevant dates, including periods of recruitment, exposure, follow-up, and data collection. | Section 2.1 | Patients diagnosed at Shanghai Changzheng Hospital between January 2014 and December 2022 were included. |
| Methods: Participants | 6a | Cohort study—Give eligibility criteria, and sources and methods of selection of participants. Describe methods of follow-up. | Section 2.1; Figure 1 | Inclusion and exclusion criteria are reported, including conventional intramedullary osteosarcoma, NAC followed by surgery, available baseline MRI, and absence of distant metastasis at initial evaluation. Patient selection is summarized in Figure 1. |
| Methods: Participants | 6b | Cohort study—For matched studies, give matching criteria and number of exposed and unexposed. | Not applicable | This was not a matched cohort study. |
| Methods: Variables | 7 | Clearly define all outcomes, exposures, predictors, potential confounders, and effect modifiers. Give diagnostic criteria, if applicable. | Sections 2.1–2.4 | Diagnosis, NAC cycle groups, OS outcome, radiomic features, clinical covariates, causal estimands, and potential outcomes are defined. |
| Methods: Data sources/measurement | 8 | For each variable of interest, give sources of data and details of methods of assessment. Describe comparability of assessment methods if there is more than one group. | Sections 2.1–2.3 | Clinical data source, MRI acquisition, ROI delineation, PyRadiomics feature extraction, and LASSO feature selection are described. Imaging limitations are acknowledged. |
| Methods: Bias | 9 | Describe any efforts to address potential sources of bias. | Sections 2.2–2.4; Discussion; Limitations | Observed clinical and radiomic covariates were incorporated into causal models. The manuscript discusses unmeasured confounding, treatment-group imbalance, missing outcome information, single-reader segmentation, and scanner variability. |
| Methods: Study size | 10 | Explain how the study size was arrived at. | Section 2.1 | All eligible patients meeting the inclusion criteria during the study period were included to maximize data utilization. |
| Methods: Quantitative variables | 11 | Explain how quantitative variables were handled in the analyses. If applicable, describe groupings and why. | Sections 2.2–2.5 | NAC cycles were categorized as <4 versus ≥4 cycles; OS was dichotomized at a 2-year landmark; continuous variables were summarized and compared using the Wilcoxon rank-sum test; radiomic features were selected using LASSO. |
| Methods: Statistical methods | 12a | Describe all statistical methods, including those used to control for confounding. | Sections 2.4 and 2.5 | S-Learner, T-Learner, X-Learner, logistic regression propensity score estimation, bootstrap uncertainty estimation, and descriptive statistical tests are described. |
| Methods: Statistical methods | 12b | Describe any methods used to examine subgroups and interactions. | Not applicable | No formal subgroup or interaction analysis was performed. |
| Methods: Statistical methods | 12c | Explain how missing data were addressed. | Sections 2.2, 3.1, and Limitations | Patients with unavailable 2-year survival status were treated as having missing outcome information. No formal multiple imputation or missing-outcome sensitivity analysis was performed. |
| Methods: Statistical methods | 12d | Cohort study—If applicable, explain how loss to follow-up was addressed. | Sections 2.2, 3.1, and Limitations | Missing overall survival information at the 2-year landmark was reported, and its influence was acknowledged as a limitation. |
| Methods: Statistical methods | 12e | Describe any sensitivity analyses. | Not applicable; Discussion and Limitations | No formal sensitivity analysis was performed. The absence of formal sensitivity analysis is acknowledged in the Discussion and Limitations. |
| Results: Participants | 13a | Report numbers of individuals at each stage of study. | Section 3.1; Figure 1 | A total of 34 eligible patients were included and categorized into high-cycle (n=16) and low-cycle (n=18) groups. Participant selection is summarized in Figure 1. |
| Results: Participants | 13b | Give reasons for non-participation at each stage. | Section 2.1; Figure 1 | Eligibility criteria and exclusion criteria are described; patient selection is summarized in Figure 1. |
| Results: Participants | 13c | Consider use of a flow diagram. | Figure 1 | A flowchart illustrating patient selection and cohort construction is provided. |
| Results: Descriptive data | 14a | Give characteristics of study participants and information on exposures and potential confounders. | Section 3.1; Tables 1 and 2 | Baseline characteristics by chemotherapy cycle group and by sex are reported. |
| Results: Descriptive data | 14b | Indicate number of participants with missing data for each variable of interest. | Section 3.1; Tables 1 and 2; Limitations | Missing PFS and OS information is reported. OS was unavailable for 7 patients. |
| Results: Descriptive data | 14c | Cohort study—Summarise follow-up time. | Tables 1 and 2; Limitations | PFS and OS summary information is reported, and limited follow-up/right-censoring is discussed as a limitation. |
| Results: Outcome data | 15 | Cohort study—Report numbers of outcome events or summary measures over time. | Section 3.1; Tables 1 and 2 | Overall survival and progression-free survival summaries and missing outcome counts are reported. |
| Results: Main results | 16a | Give unadjusted and, if applicable, confounder-adjusted estimates and their precision. | Table 4; Section 3.3 | Exploratory ATE estimates and 95% confidence intervals from causal meta-learners are reported. |
| Results: Main results | 16b | Report category boundaries when continuous variables were categorized. | Section 2.2 | NAC cycles were categorized as <4 versus ≥4 cycles; OS was dichotomized at the 2-year landmark. |
| Results: Main results | 16c | If relevant, consider translating estimates of relative risk into absolute risk for a meaningful time period. | Not applicable | Treatment effects were reported as probability differences, not relative risks. |
| Results: Other analyses | 17 | Report other analyses done, e.g., subgroup/interactions/sensitivity analyses. | Sections 3.2 and 3.3; Figure 3; Table 5 | Radiomic feature selection and group-wise comparisons are reported. Table 5 provides illustrative ITE examples. No formal sensitivity analysis was performed. |
| Discussion: Key results | 18 | Summarise key results with reference to study objectives. | Discussion | The Discussion summarizes the feasibility of the framework, instability of ATE estimates, and exploratory interpretation. |
| Discussion: Limitations | 19 | Discuss limitations, taking into account sources of potential bias or imprecision; discuss direction and magnitude of potential bias. | Discussion; Limitations | The manuscript discusses small sample size, inconsistent learner results, missing outcome information, sex imbalance, unmeasured confounding, dichotomization of OS, single-reader segmentation, lack of ICC, T1-only radiomics, scanner variability, and limited generalizability. |
| Discussion: Interpretation | 20 | Give a cautious overall interpretation of results considering objectives, limitations, multiplicity, similar studies, and other evidence. | Discussion; Conclusion | Findings are interpreted as methodological and hypothesis-generating rather than clinically actionable. |
| Discussion: Generalisability | 21 | Discuss the generalisability (external validity) of the study results. | Discussion; Limitations; Conclusion | The manuscript states that results require validation in larger multicenter cohorts and may not generalize beyond conventional intramedullary osteosarcoma. |
| Other information: Funding | 22 | Give the source of funding and the role of funders. | Declarations, Funding | Funding sources are listed in the Funding section. |

*Note: Page numbers may be updated after final journal formatting. Section, table, and figure locations are provided to identify where each item is reported in the revised manuscript.*
