# Supplementary material for: Radiomics-based causal machine learning for exploratory treatment-effect estimation of neoadjuvant chemotherapy cycle intensity in osteosarcoma: a proof-of-concept study
Source: BMC Med Imaging. 2026 Jun 30;26:467. doi: 10.1186/s12880-026-02533-7 (PMC13599299; doi:10.1186/s12880-026-02533-7)
Supplement: Supplementary file 2 [file 12880_2026_2533_MOESM2_ESM.docx]

**TRIPOD Checklist: Prediction Model Development**

Radiomics-Based Causal Machine Learning for Exploratory Treatment-Effect Estimation of Neoadjuvant Chemotherapy Cycle Intensity in Osteosarcoma: A Proof-of-Concept Study

| **Section/Topic** | **Item** | **Checklist Item** | **Location in manuscript** |
| --- | --- | --- | --- |
| Title and abstract | 1 | Identify the study as developing and/or validating a multivariable prediction model, the target population, and the outcome to be predicted. | Reported in: Title. The study is identified as a radiomics-based causal machine learning proof-of-concept study in osteosarcoma, focused on exploratory treatment-effect estimation for survival outcome. |
| Title and abstract | 2 | Provide a summary of objectives, study design, setting, participants, sample size, predictors, outcome, statistical analysis, results, and conclusions. | Reported in: Abstract. The abstract summarizes the objective, retrospective single-center design, sample size (n=34), predictors/covariates, outcome, causal meta-learners, exploratory results, and cautious conclusion. |
| Introduction | 3a | Explain the medical context (including whether diagnostic or prognostic) and rationale for developing or validating the multivariable prediction model, including references to existing models. | Reported in: Section 1 (Background). The medical context of osteosarcoma, NAC cycle uncertainty, radiomics, and causal machine learning is described with relevant references. |
| Introduction | 3b | Specify the objectives, including whether the study describes the development or validation of the model or both. | Reported in: Section 1 (final paragraph). The study describes development of an exploratory proof-of-concept causal meta-learning framework; no external validation or deployable clinical model was intended. |
| Methods | 4a | Describe the study design or source of data (e.g., randomized trial, cohort, or registry data), separately for the development and validation data sets, if applicable. | Reported in: Section 2.1. The study used retrospective single-center cohort data from Changzheng Hospital. |
| Methods | 4b | Specify the key study dates, including start of accrual; end of accrual; and, if applicable, end of follow-up. | Reported in: Section 2.1. Patients diagnosed between January 2014 and December 2022 were included. |
| Methods | 5a | Specify key elements of the study setting (e.g., primary care, secondary care, general population) including number and location of centres. | Reported in: Section 2.1. The study was conducted at a single hospital center, Changzheng Hospital, Shanghai. |
| Methods | 5b | Describe eligibility criteria for participants. | Reported in: Section 2.1. Inclusion and exclusion criteria are described, including conventional intramedullary osteosarcoma, NAC followed by surgery, baseline MRI, and absence of distant metastasis. |
| Methods | 5c | Give details of treatments received, if relevant. | Reported in: Section 2.2. Treatment was defined according to NAC cycle intensity: low-cycle (<4 cycles) and high-cycle (>=4 cycles). |
| Methods | 6a | Clearly define the outcome that is predicted by the prediction model, including how and when assessed. | Reported in: Section 2.2. The outcome was overall survival dichotomized at the 2-year landmark, assessed from diagnosis to death from any cause. |
| Methods | 6b | Report any actions to blind assessment of the outcome to be predicted. | Not applicable / not performed. Outcome data were obtained retrospectively from clinical records; no blinded outcome adjudication procedure was reported. |
| Methods | 7a | Clearly define all predictors used in developing or validating the multivariable prediction model, including how and when they were measured. | Reported in: Sections 2.3 and 2.4. Predictors/covariates included baseline clinical variables and selected MRI-derived radiomic features; MRI was acquired before NAC. |
| Methods | 7b | Report any actions to blind assessment of predictors for the outcome and other predictors. | Not applicable / not performed. Predictor extraction and modeling were retrospective; no formal blinding procedure for predictors was reported. ROI delineation was performed by a single reader and acknowledged as a limitation. |
| Methods | 8 | Explain how the study size was arrived at. | Reported in: Section 2.1. All eligible patients during the study period were included to maximize data utilization because of the small cohort size. |
| Methods | 9 | Describe how missing data were handled (e.g., complete-case analysis, single imputation, multiple imputation) with details of any imputation method. | Reported in: Sections 2.2, 3.1, and Limitations. Missing 2-year OS information was reported descriptively; no formal multiple imputation or missing-outcome sensitivity analysis was performed. |
| Methods | 10a | Describe how predictors were handled in the analyses. | Reported in: Sections 2.3-2.5. Radiomic features were extracted with PyRadiomics, selected using LASSO with ten-fold cross-validation, and integrated with clinical variables. |
| Methods | 10b | Specify type of model, all model-building procedures (including any predictor selection), and method for internal validation. | Reported in: Sections 2.3 and 2.4. Feature selection used LASSO; treatment effects were estimated using S-Learner, T-Learner, and X-Learner with AutoGluon; bootstrap was used for ATE uncertainty. Internal 8:2 split was used only as a technical check. |
| Methods | 10d | Specify all measures used to assess model performance and, if relevant, to compare multiple models. | Reported in: Section 3.3 and Table 3. AUC, F1 score, accuracy, and precision were reported as descriptive technical outputs, not as evidence of validity or generalizability. |
| Methods | 11 | Provide details on how risk groups were created, if done. | Not applicable. No clinical risk groups were created. Treatment-cycle groups were prespecified as <4 vs >=4 NAC cycles (Section 2.2). |
| Results | 13a | Describe the flow of participants through the study, including the number of participants with and without the outcome and, if applicable, a summary of the follow-up time. A diagram may be helpful. | Reported in: Section 3.1 and Figure 1. Participant selection and cohort construction are summarized. |
| Results | 13b | Describe the characteristics of the participants (basic demographics, clinical features, available predictors), including the number of participants with missing data for predictors and outcome. | Reported in: Tables 1 and 2 and Section 3.1. Baseline demographic and clinical characteristics and missing outcome information are reported. |
| Results | 14a | Specify the number of participants and outcome events in each analysis. | Reported in: Sections 3.1-3.3 and Tables 1-4. Analyses used 34 patients; treatment groups included 16 high-cycle and 18 low-cycle patients. Missing 2-year OS status is reported. |
| Results | 14b | If done, report the unadjusted association between each candidate predictor and outcome. | Reported in: Section 3.2 and Figure 3. Group-wise comparisons of selected radiomic and clinical variables are presented where applicable. |
| Results | 15a | Present the full prediction model to allow predictions for individuals (i.e., all regression coefficients, and model intercept or baseline survival at a given time point). | Partially applicable. No deployable prediction formula is provided because AutoGluon-based causal meta-learners were used for exploratory treatment-effect estimation rather than a validated clinical prediction model. This limitation is acknowledged in Discussion/Limitations. |
| Results | 15b | Explain how to the use the prediction model. | Partially applicable. Table 5 illustrates interpretation of model-derived ITE outputs, but the manuscript explicitly states that these examples are not treatment recommendations. |
| Results | 16 | Report performance measures (with CIs) for the prediction model. | Reported in: Section 3.3 and Tables 3-4. Model performance metrics and ATE estimates with 95% confidence intervals are reported, with caution that they are exploratory. |
| Discussion | 18 | Discuss any limitations of the study (such as nonrepresentative sample, few events per predictor, missing data). | Reported in: Discussion and Limitations. Limitations include small sample size, overfitting/model instability, missing outcome data, treatment-group imbalance, unmeasured confounding, single-reader ROI delineation without ICC, T1-only radiomics, and lack of external validation. |
| Discussion | 19b | Give an overall interpretation of the results, considering objectives, limitations, and results from similar studies, and other relevant evidence. | Reported in: Discussion and Conclusion. Results are interpreted as proof-of-concept and hypothesis-generating rather than clinically actionable. |
| Discussion | 20 | Discuss the potential clinical use of the model and implications for future research. | Reported in: Discussion and Conclusion. The manuscript states that the framework requires validation in larger multicenter cohorts with standardized imaging, adequate event counts, longer follow-up, and time-to-event causal modeling before clinical utility can be assessed. |
| Other information | 21 | Provide information about the availability of supplementary resources, such as study protocol, Web calculator, and data sets. | Reported in: Declarations (Availability of data and materials) and Methods. Data are available from the corresponding author on reasonable request due to privacy restrictions; analysis scripts were version-controlled. No web calculator or deployable model is provided. |
| Other information | 22 | Give the source of funding and the role of the funders for the present study. | Reported in: Declarations (Funding). Funding sources are listed. |

*Note: Section/table/figure locations are provided because final page numbers may change during typesetting.*
